# Regional Chemotherapy Is a Valuable Second-Line Approach in Metastatic Esophageal Cancer after Failure to First-Line Palliative Treatment

**DOI:** 10.3390/curroncol29070386

**Published:** 2022-07-11

**Authors:** Yogesh Vashist, Kornelia Aigner, Miriam Dam, Sabine Gailhofer, Karl R. Aigner

**Affiliations:** 1 Clinic for Surgical Oncology, Medias Klinikum Burghausen, Krankenhausstrasse 3a, 84489 Burghausen, Germany; m.dam@medias-klinikum.de (M.D.); s.gailhofer@medias-klinikum.de (S.G.); info@prof-aigner.de (K.R.A.); 2 Department of Tumor Biology, Medias Klinikum Burghausen, Krankenhausstrasse 3a, 84489 Burghausen, Germany; kornelia.aigner@medias-klinikum.de

**Keywords:** esophageal cancer, survival, second-line therapy, regional chemotherapy, isolated thoracic perfusion, arterial infusion

## Abstract

Background: Therapeutic options in metastatic esophageal cancer (EC) are limited with unsatisfactory results. We evaluated the efficacy of regional chemotherapy (RegCTx) approach in diffuse metastatic EC using arterial infusion (AI), upper abdominal perfusion (UAP) and isolated-thoracic perfusion (ITP) in 14 patients (N = 8 adenocarcinoma (AC) and N = 6 squamous cell carcinoma (SQCC)) after failure to first-line palliative treatment. Methods: All patients had previously failed first-line palliative treatment attempt with systemic chemotherapy (sCTx). In total 51 RegCTx cycles (12 AI, 3 UAP and 36 ITP) were applied using cisplatin, Adriamycin and Mitomycin C. The outcome was evaluated using RECIST criteria with MediasStat 28.5.14 and SPSS–28.0. Results: No grade III or IV hematological complications occurred. The overall response rate was 41% partial response, 27% stable and 32% progressive disease. Median overall survival (OS) was 38 months (95%CI 10.1–65.9). The OS was better in SQCC with 51 months The RegCTx specific survival was 13 months (95%CI 2.9–23.1) in the entire cohort and 25 months in SQCC patients. Conclusion: RegCTx is a valuable safe approach and superior to the current proposed therapeutic options in metastatic EC after failure to first-line therapy.

## 1. Introduction

Esophageal cancer (EC) represents the sixth most common cause of cancer related death and ranks ninth in the most common cancer globally. Approximately ≥50% of the patients are diagnosed with stage IV disease and recurrence after primary curative intended therapy is also common. Hence the overall five-year survival rate of recurrent or metastatic disease remains below 5% [1,2,3,4,5].

Unfortunately, recommendations for the management of recurrent and metastatic disease are scare even in the national and oncological society guidelines [6,7,8]. Although within the last few years many trials have been conducted such as Keynote-181 and ATTRACTION-3 trial as well as phase III studies with irinotecan, docetaxel and Ramucirumab, the overall outcome remains highly unsatisfactory with ≤10 months of survival in patients failing to respond to first-line treatment [4,9,10,11]. This is even of more concern taking the quality of life (QoL) and required duration of therapy into consideration.

The regional chemotherapy (RegCTx) approach is an oncological approach with very low toxicity profile and high tumor response due to high cytotoxic drug concentrations in an isolated perfusion bed [12,13,14,15,16,17,18,19]. In addition, the therapy can be focused on limited regions if necessary, using the same technique (e.g., upper abdominal perfusion (UAP), isolated thoracic perfusion (ITP), and intra-arterial infusion (AI)). The restricted perfusion bed that is treated during RegCTx allows potentiation of drug concentration levels at the tumor site compared to sCTx despite using up to 20–50% of lower overall cytotoxic drug amount. Furthermore, the possibility to perform a chemo-filtration ensures lowest systemic toxicity effects [12,14,15,16,19]. RegCTx efficacy has been proven in many cancers including metastatic gastric cancer but has not been reported in advanced metastatic EC.

Here we report on our institutional experience with 14 advanced metastatic EC patients undergoing RegCTx after failure to first-line palliative treatment.

## 2. Patients and Methods

### 2.1. Characterization of the Study Population

The study was approved by the Medical Ethical Committee Medias Clinic, Burghausen, Germany (MIRB20211108) and informed consent was obtained from all subjects involved in the study. In total 14 patients with metastatic EC were enrolled in this study. Patients were treated between 2002 and 2019. RegCTx is associated with very low morbidity and mortality and the possibility to combine infusion and perfusion techniques add to the fact that even patients with ECOG upto 3 could be treated in this series. Clear exclusion criteria were severely impaired cardio-pulmonary, liver and renal function, ongoing infection and vascular anatomy not allowing a safe access. Table 1 depicts all characteristics of the patients. All patients presented with highly advanced metastatic EC out of which five (35%) had a documented resection of the primary tumor and presented now with a diffuse relapse of the disease. Previous therapy in the cohort consisted of sCTx in all 14 patients. Radiation therapy was documented in eight (57%) patients. Seven (50%) of the patients had received both sCTx and radiation. In addition, two patients had an implanted esophageal stent and two patients underwent metastasectomy (cerebral and pulmonary) along with radiation after relapse of the disease. The applied chemotherapy regime displayed a broad spectrum with taxane, fluorouracil, oxaliplatin, epirubicin, capecitabine, and in two cases also Herceptin.

Metastatic sites included primarily the various lymph node stations in the abdomen, thorax, retroperitoneum and cervical region and lungs followed by liver. Three (21%) patients also presented with a local relapse along with other metastatic sites.

### 2.2. Cytotoxic Drugs and Methods

#### 2.2.1. Regional Chemotherapy Techniques

**Figure 1:**
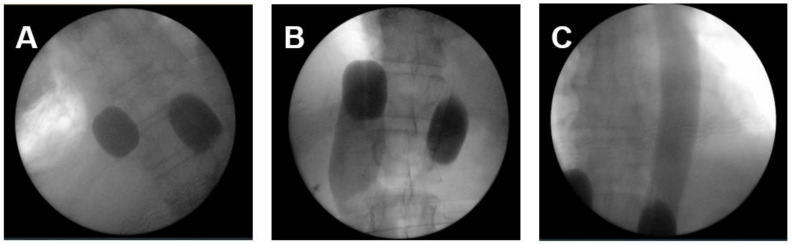
The intraoperative pictures demonstrate (**A**) placement of balloon catheter and level of blockage in the inferior cava vein and aorta; (**B**) blood flow stop below the right atrium from downwards and depiction of inferior cava vein and hepatic veins; (**C**) blood flow stop below the inflated balloon catheter in the aorta and depiction of covering entire thoracic aorta with pulsatile injections.

The applied perfusion techniques have been described in detail previously [14,19,20]. To address the primary tumor, mediastinal, lung and cervical metastases an ITP was performed. In total 36 ITPs were conducted. Briefly, RegCTx was performed under general anesthesia and full systemic anticoagulation with heparin. An approximately five cm incision was placed in the groin region and the femoral vessels explored. The vessels were looped with vessel-loops and a venous and arterial three-channel stop-flow balloon catheter was inserted in the inferior vena cava and aorta. Cava catheter was blocked beneath the right atrium, and the aorta was blocked slightly below of the diaphragm plane. Placement was undertaken only under X-ray control. Correct placement of the catheters was documented by cavography and aortography, respectively (Figure 1A–C). The upper arms were blocked by pneumatic cuffs ensuring the isolation of the chest, head, and neck area. Chemotherapy was applied manually with high pulsatile pressure against the aortic blood stream through the coaxial channel of the arterial balloon catheter.

After chemotherapy administration, autochthonous perfusion with the heart as internal pump with blocked stop flow catheters was maintained for 15 min.

After 15 min of total perfusion time the balloons were deflated and chemo-filtration started until five liters of substitutional volume was reached. Drugs were chosen according to their cytotoxic potential.

To address the hepatic and abdominal tumor load UAP was conducted in three (6%) patients. For the UAP, cuffs were placed around both thighs and in the first step, the venous balloon was positioned right beneath the diaphragm and the arterial balloon was placed right beneath the celiac trunk. After angiographic verification of the celiac trunk perfusion the chemotherapeutic drugs were infused during one minute from the tip of the arterial balloon ensuring the entire cytotoxic drug is spread through the celiac perfusion bed. Parallel to this the venous balloon was inflated. Thereafter the arterial balloon was immediately slipped upstream and positioned above the celiac trunk ensuring mesenterial stopped blood flow phase with very high drug concentrations in the upper abdomen as a consequence of this little change in the arterial catheter position. The stopped blood flow phase lasts for the first five minutes.

For step two, the perfusion was run through side holes in the catheter tubes downstream the arterial and venous balloons. This resulted in still relatively high drug concentrations in the whole abdominal perfusion bed under hypoxic condition for five minutes. The UAP was also combined with chemo-filtration as described above.

In patients in whom potential strong tumor necrosis post perfusion was feared with sepsis, AI was applied only or prior to UAP. For this, an angiographic sidewinder catheter was inserted via the femoral artery into the celiac trunk or hepatic artery. Drugs were infused as short infusions during five to 12 min with short term plateau of considerably high drug concentrations in the perfusion bed. Twelve cycles have been applied as AI combined with full anesthesia and followed by chemo-filtration.

#### 2.2.2. Cytotoxic Drugs

For the treatment Cisplatin, Adriamycin, and Mitomycin C were used. Drug dosages for perfusions were 50–100 mg Cisplatin, 20–50 mg Adriamycin (cumulative maximum dose up to 600 mg), and 10–20 mg Mitomycin c (cumulative maximum dose up to 60 mg), respectively. Intra-arterial infusions have been conducted with 30–40 mg Cisplatin alone or with 10–30 mg Adriamycin, and 10–20 mg Mitomycin. Selection of cytotoxic drugs was based upon institutional experience and the limited data from experimental in-vitro cell culture studies that demonstrated for mitomycin C and doxorubicin an increased cell toxicity under hypoxic conditions and cisplatin equal cell toxicity under aerobic and hypoxic conditions [21].

#### 2.2.3. Treatment Cycles

Regional chemotherapy has been applied in treatment cycles. Each treatment cycle consisted of either one isolated perfusion or one intra-arterial infusion followed by chemo-filtration. Each therapy cycle was followed by a three-week therapy free interval.

In total, 51 cycles were applied consisting of 36 ITP, 3 UAP and 12 AI. In nine (64%) patients only a single RegCTx technique was applied whereas in the remaining five a combination of two techniques was applied over the course of the entire treatment period. Minimum of two cycles were applied in 86% of the patients. The techniques were alternated for different cycles for each patient if different metastatic locations were to be treated.

### 2.3. Statistical Analysis

For statistical analysis, SPSS for Windows (Version 28.0) was used. RegCTx specific survival and overall survival (OS) curves of the patients were plotted by using the Kaplan–Meier method and analyzed using the log-rank test. Significant statements refer to *p*-values of two-tailed tests that were *p* < 0.05. Results are presented as median survival in months with 95% confidence interval (CI) or if median was not reached with mean values. The OS was computed as the time period from the date of first diagnosis to the date of death or last follow-up, whichever occurred first. The RegCTx specific survival was defined as the time period from the date of first RegCTx to last follow-up or date of death, whichever occurred first. Patients alive at the last follow-up date were censored.

The response evaluation under RECIST criteria was undertaken with the MediasStat software version 28.5.14 and addressed as partial response (PR), stable disease (SD) or progressive disease (PD). In addition, quality of life (QoL) was assessed based on an institution specific questionnaire including nausea, vomiting, hair loss, diarrhea, mucosal changes, fatigue, exhaustion and loss of appetite [22]. QoL was assessed during the in-hospital stay pre and post RegCTx treatment.

The data that support the findings of this study are available from the authors upon reasonable request.

## 3. Results

No RegCTx associated mortality occurred. The overall morbidity rate was 57% with being dominated by the development of lymph fistulas at the inguinal access site, N = 8 (57%) patients, over the entire treatment duration. However, all fistulas were treated successfully conservatively. Due to the development of wound hematoma, one patient required an operative wound revision. Incidence of general side effects such as nausea and fatigue have been very low and only mild and did not require any additional medication to post RegCTx standard protocol. Hair loss, hand-foot syndrome, and neuropathy did not occur. No grade three or four hematological complication occurred.

**Figure 2:**
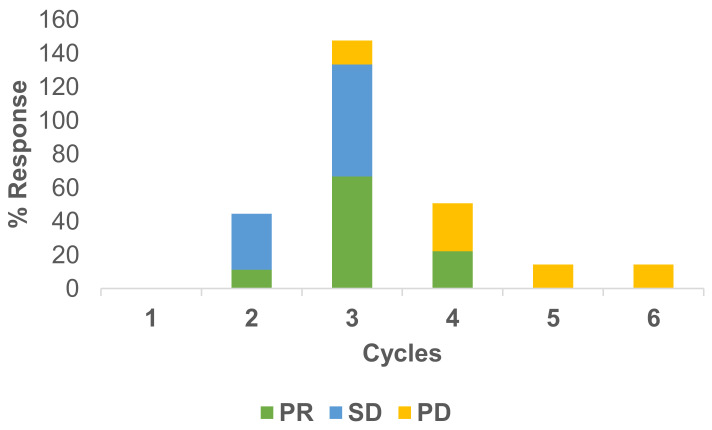
Cycle stratified response to regional chemotherapy. PR and SD are more common during the earlier cycles compared to PD which is more frequent at later cycles. The best response appears after the third cycle.

Responses to the treatment have been measured under RECIST criteria. Usually, after two cycles of therapy, a CT scan has been conducted. In total, 22 scans were available for response evaluation. The overall response rate was 41% PR, 27% SD and 32% PD for the RegCTx in the entire cohort and over the entire period of follow-up. Cycle specific response demonstrated best PR and SD after the third cycle and both being declining substantially afterwards. On the other hand, PD was less frequent in the earlier cycles but picked up after the third cycle. Figure 2 depicts the response evaluation.

The institute specific QoL indicator as a clinical response parameter was documented during the in-hospital stay pre and post RegCTx treatment. The overall clinical response (QoL) evaluation yielded CR in 9%, PR in 31%, SD in 41% and PD in 19% of the patients.

The median OS was 38 months (95%CI 10.1–65.9) with 8 (57%) patients having a survival lasting ≥22 months. Survival rates reached 79%, 50%, and 35% at years one, two, and three, respectively.

**Figure 3:**
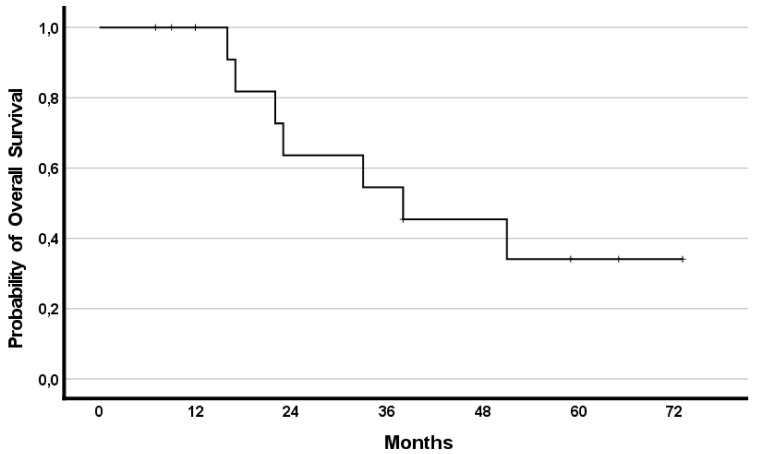
Cumulative overall survival in the entire cohort of 14 patients.

**Figure 4:**
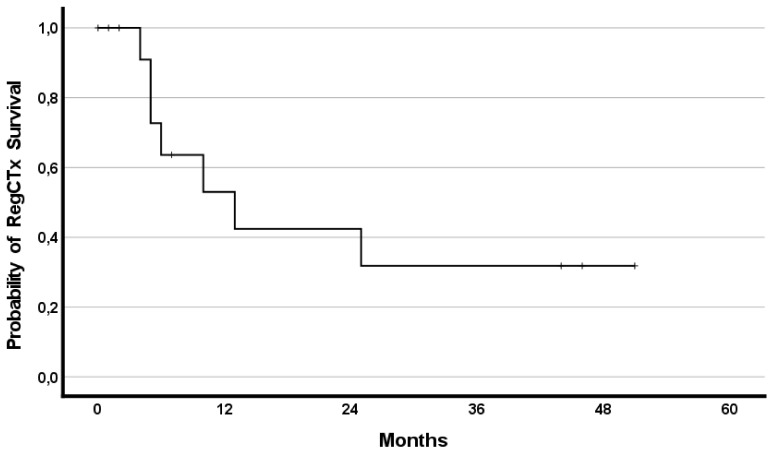
Cumulative regional chemotherapy specific survival in the entire cohort of 14 patients.

In addition, the effect of RegCTx resulted in a median survival benefit, from the timepoint of RegCTx initiation, of 13 months (95%CI 2.9–23.1) and 4 (29%) patients being alive even after 12 months under the RegCTx. The according survival rates were 29%, 21% and 21% at years one, two, and three, respectively. Figure 3 and Figure 4 demonstrate the OS and RegCTx specific survival in all 14 patients.

Sub-analysis stratified upon underlying histological subtype demonstrated a median OS benefit of 23 months (95%CI 0–48.2) for AC and 51 months (95%CI 12.3–89.6) for SQCC (*p* = 0.521), respectively. Furthermore, patients who had previously undergone resection of the primary tumor and presented with a diffuse relapse displayed a better survival tendency with median survival not reached but mean OS of 57.1 (95%CI 39.5–74.8) compared to median OS of 22months (95% CI 2.8–41.2; *p* = 0.09) in patients with primary diffuse metastatic EC.

## 4. Discussion

Curative intended management of EC is highly complex with regard to surgical techniques, sCTx combination and radiation dose as well as the sequence of therapeutical cascade [6,7,23]. In contrast the management of metastatic and recurrent EC is poorly defined but chaotic and even in the national and oncological society guidelines not many options are proposed. Besides the treatment is oriented to the management of metastatic gastric cancer also [4,8,24].

We have recently published our experience with the efficacy of RegCTx in metastatic gastric cancer [14]. Here we report on our experience with RegCTx approach in metastatic EC patients after failure to first-line treatment. In a cohort of 14 patients with advanced metastatic EC patients, of whom 5 (36%) had previously undergone resection of the primary tumor and all with failed first-line treatment and a progressive disease, displayed with the RegCTx, as a safe and viable approach, a superior outcome with RegCTx specific survival of 13 months compared to the other current available second-line therapy regimes in metastatic EC.

Systemic chemotherapy, with platinum and fluoropyrimidine as a doublet combination, builds the cornerstone for the management of metastatic EC [25]. For SQCC several studies from Asian countries demonstrated response rates of 30% and median OS between six to ten months with cisplatin and 5-FU [26,27,28,29]. For AC in European countries prior to initiation of a sCTx the Her2-status evaluation is mandatory [6]. At present for HER-2 negative tumors a combination (“doublet”) sCTx with platin and fluoropyrimidine is recommended. However, the different available randomized clinical trials from around the world comparing various combination of chemotherapeutic agents have only demonstrated a median OS of less than one year with grade 3 and 4 toxicity profiles in at least >50% but also reaching up to 82% of the patients [30,31,32,33]. Data for HER2-positive tumors from the ToGA trial imply a benefit for Trastuzumab addition to the systemic component of 5 months. However, it needs to be pointed out that in the ToGA trial 80% of the patients had gastric cancer and only 20% were gastro-esophageal-junction cancer. Furthermore, the ToGA trial applies to AC only and in addition, efficacy of Trastuzumab plus chemotherapy is only substantially improved in patients with high expression of HER2 protein (immunohistochemistry 2+ and FISH positive or immunohistochemistry 3+) compared with patients with low expression of HER2 protein (immunohistochemistry 0 or 1+ and FISH positive) [34].

The lack of specific recommendations for the treatment of metastatic EC up to recently is in line with the first-line treatment of our patient cohort with a widely diverse chemotherapeutic agents utilized and indicates towards a representative EC cohort with failure to first-line treatment.

Second-line treatment for metastatic AC subtype is recommended in general without specifically naming particular regimens [6,35,36,37,38,39]. For SQCC however, the recommendation is more of spongious character only. The available studies so far including docetaxel, irinotecan and or Ramucirumab reported a survival benefit of less than five months compared to best supportive care only [35,36,37]. This also pinpoints towards the efficacy of RegCTx in metastatic EC with an RegCTx specific survival of 13 months.

Meanwhile few newer studies are available. The ATTRACTION-3 trial comparing nivolumab with taxane after failure to previous doublet sCTx demonstrated an OS of almost 10.8 months with nivolumab compared to eight months with taxane. However, 18% of the patients in the nivolumab group had grade 3 or 4 treatment-related adverse events compared with 63% in the sCTX group [10].

The recently published KEYNOTE-181 trial reported on pembrolizumab compared to sCTx (taxane or irinotecan) in the second-line setting with median OS of 9.3 months versus 6.7 months is in line in terms of outcome and grade 3–5 complication rate with the ATTRACTION-3 trial [9]. However, both trials do represent the current standard of care in the second-line setting for metastatic EC with SQCC histological subtype. For AC patients after failing to respond to the first-line treatment paclitaxel monotherapy or combined treatment with Ramucirumab are used in clinical practice according to the results of the RAINBOW and KEYNOTE-061 phase III trials [11,40].

None of the patient with RegCTx in our cohort suffered a grade 3 or higher systemic side effect and the outcome taking the RegCTx specific survival of 13 months into consideration pinpoints towards a safer and more superior option in the second-line setting of metastatic EC.

Another aspect to be considered in the management of these highly compromised patients is the duration of therapy. Systemic combinations are recommended for up to 24 weeks and for Transtuzumab, Ravicurimab, and Pembrolizumab, a maintenance therapy is even recommended [41,42].

In contrast, our therapy approach has required a hospital stay for RegCTx of only a few days (up to five days) per cycle and 86% of our patients received at least two cycles. Our data demonstrate that the first three cycles are the most efficient cycles with the best documented disease response. In our cohort, patients were able to move around on the day of RegCTx and no documented side effect appeared that required re-hospitalization. Patients could be discharged after each cycle within one week from the hospital followed by a three-week therapy free interval. Since QoL is a major aspect in the management of these patients our approach ensured no impairment in QoL but a significant improvement. Taken into consideration that we have treated mostly ECOG 2 and 3 patients, this pinpoints towards justification of this approach without even taking the OS benefit and low toxicity profile into account.

It has to be outlined that all of our patients were not sCTx naive but had failed to first-line treatment and did present with a progress of the disease. Hence, also in terms of the oncological outcome our data indicate towards a beneficial role of RegCTx in metastatic EC with a median OS of 38 and RegCTx specific survival of 13 months.

The reason for low toxicity profile with our RegCTx approach is based upon the frequent combination of RegCTx with chemo-filtration on the one hand but also using fewer total dosages and directed therapy towards a limited perfusion bed to reduce collateral damage to other organs hence also reducing the cumulative toxicity over time with ensuring a longer treatment phase if required [12,14,17,20,43].

Limitations of the current study are the long inclusion period, limited number of patients, heterogenous group with regard to pre-treatment, and non-standardized QoL assessment.

Besides the RECIST response evaluation, we have also mentioned the QoL estimation based on a subjective questionnaire (patient own remarks) [22]. This is clearly not objective or reported in a standardized manner and should be assessed with a formal QoL questionnaire in future.

The defined role of second-line therapy in metastatic EC is only emerging slowly over time hence few patients might have had a better survival if those options would have been available at given time. However, we have been able to demonstrate that after first-line therapy failure a clear indisputable benefit with RegCTx can be achieved. In addition, we have only included EC patients in our study cohort and intentionally excluded gastric or gastro-esophageal-junction cancer hence we present a clinically homogeneous metastatic cohort. However, the pre-treatment regime of the included patients is widely spread but represents the clinical practice up to now in general. Furthermore, RegCTx is a highly individualized concept, and hence adaptation with regard to form and frequency is essential and also dependent on patient’s own will to continue or terminate the therapy.

In conclusion, RegCTx offers a safe and low toxicity associated therapy approach in highly advanced metastatic EC patients failing to respond to first-line therapy but clearly benefitting from RegCTx. Future studies are required to evaluate the role of RegCTx in the multimodal management of metastatic EC in the first-line and also neoadjuvant setting. Combination of RegCTx with newly available immune checkpoint inhibitors is also warranted.

## Figures and Tables

**Table 1:** All characteristics of the patients.

Patient’s Characteristics		
Variable	N	%
All	14	100
Age, median years; range	55	35–68
Sex		
female	2	14.3
male	12	85.7
Primary metastatic	9	64.3
Relapse	5	35.7
Metastatic site		
liver	5	35.7
lymph nodes	8	57.1
lungs	8	57.1
bone	2	14.3
brain	2	14.3
others	2	14.3
local relapse	3	21.4
Previous therapy		
Resection primary tumor	5	35.7
Systemic chemotherapy	14	100
Radiotherapy	8	57.1
Stent	2	14.3
Metastasectomy	2	14.3
Regional chemotherapy		
Total Cycles	51	100
Art. Infusion	12	23.5
UAP	3	5.8
ITP	36	70.6

## Data Availability

The data presented in this study are available in the article.
